# Potential Prebiotic Properties of Nuts and Edible Seeds and Their Relationship to Obesity

**DOI:** 10.3390/nu10111645

**Published:** 2018-11-03

**Authors:** Clara S. A. Sugizaki, Maria Margareth V. Naves

**Affiliations:** School of Nutrition, Federal University of Goiás, Rua 227, s/n, St Leste Universitário, Goiânia, Goiás 74605-080, Brazil; clarasugizaki@gmail.com

**Keywords:** nuts, seeds, polyphenols, dietary fiber, microbiota, dysbiosis, overweight, obesity, health benefits

## Abstract

Obesity is a global epidemic chronic condition and is progressing at a rapid rate. This review focuses on the potential prebiotic properties of nuts and edible seeds and the plausible mechanisms that their consumption may help the prevention and the management of overweight and obesity. The literature review was performed by searching papers about the topic in MEDLINE and SCOPUS databases. The healthy attributes of nuts and edible seeds, especially dietary fibers and polyphenols contents, indicate that their mechanism of weight gain prevention may occur through interaction with the gut microbiota, by means of prebiotic effects. Among the etiological factors associated with obesity, the gut microbiota seems to play a significant role. Dysbiosis causes an imbalance in energy homeostasis that contributes to obesity. Three mechanisms are proposed in this review to explain the potential role of nut and edible seed consumption on intestinal homeostasis and body weight control: maintenance of the enteric barrier integrity, improvement of anti-inflammatory status and enhancement of butyrate synthesis. Further high-quality clinical trials should explore the interaction between oilseed consumption, microbiota, and body adiposity control, particularly investigating the microbiota metabolites and their relation to the prevention and management of obesity.

## 1. Introduction

Overweight and obesity are regarded as a public health priority, because of their pandemic progression with more than two billion people affected worldwide, representing about 30% of the world population, and owing to its potential comorbidity [1]. Obesity is a complex disease with a multiple etiology, including genetic predisposition, physiological mechanisms, psychological factors, physical activity pattern, and environmental and social contexts. Nonetheless, this disorder is strongly related to dietary habits. Rapidly increasing rates of overweight and obesity in most countries indicate that the primary determinant of obesity development comes from behavioral and dietary changes [2].

Calorie intake control is recognized as the primary strategy for addressing excess body weight. In this perspective, there is a tendency to avoid nut consumption in the prevention and treatment of obesity because of their high energy value. However, according to the epidemiological evidence, nut consumption has strong inverse association with obesity and may confer beneficial effects on metabolic risk factors [3,4,5]. A meta-analysis including 47 randomized controlled trials with 2211 healthy or dyslipidemic participants concluded that the diets with nut supplementation didn’t increase adiposity, based on body weight, body fat, and waist circumference, compared to control diets [6]. Furthermore, clinical trials demonstrated that including nuts as part of a weight-loss regimen can lead to greater weight loss than simply following a low-fat diet [7,8,9,10,11,12].

The potential beneficial effects of nut consumption in prevention and management of overweight and obesity are related to their content in nutrients and bioactive compounds. Oilseeds have high lipid, protein, and fiber contents, but low digestible carbohydrates levels, and these attributes have been associated with decreased glucose and insulin peaks, low glycemic index, and high satiety [13,14]. In addition, nuts and edible seeds contain high amounts of monounsaturated fatty acids (MUFA), especially hazelnut [15], and they are also a source of lipophilic compounds, as tocopherols, mainly almond and hazelnut [16], and pistachio stands out for its high contents of phytosterols and carotenoids [17]. Phenolic compounds are the most abundant phytochemicals in nuts and edible seeds [16], especially flavonoids and tannins, largely found in walnut and pecan [15,17].

The diversity of compounds found in nuts and edible seeds are related to their antioxidant and anti-inflammatory properties and to several health benefits, including the possible effects on the remodeling of gut microbiota [6,14,18]. The fiber and polyphenols content of nuts may modulate the gut microbiota profile, aiding intestinal homeostasis, increasing butyrate synthesis, and maintaining the enteric barrier integrity, generating anti-inflammatory effects [19,20]. Therefore, nut and edible seed consumption may be adjuvant for the management of obesity and other inflammatory diseases [18]. This review focuses on the potential prebiotic properties of nuts and edible seeds and the plausible mechanisms that may explain the beneficial effects of their consumption on the prevention and management of overweight and obesity.

## 2. Materials and Methods

The literature review was carried out from July to December 2017 and updated in July–September 2018, according to methodological criteria as described in the following. The literature search was conducted in MEDLINE and SCOPUS databases. The following combination of Medical Subject Headings (MeSH terms) were used: “nuts OR seeds AND obesity”, “nuts OR seeds AND microbiota”, “nuts OR seeds AND obesity AND microbiota”, “microbiota AND polyphenols”, “nuts OR seeds AND health benefits”, “nuts OR seeds AND *Lactobacillus* (OR *Bifidobacterium*)”. All terms were combined with the filter for studies published up to the last ten years, except for relevant papers in this field, and for studies using animal models and human trials, but some in vitro studies were included to reinforce the in vivo evidence. The relevance of the publications was based on the titles, abstracts, and impact factor above 1.5 (from Journal of Citation Reports, Web of Knowledge), except for three papers related to baru almond, an edible seed native to Brazil. Studies with polyphenols from other plants, medicinal compounds, or outcomes not related to obesity were excluded. Publications in languages other than English were also excluded. Additional clinical trials were selected by carefully reading the references of the selected articles.

## 3. Relationship between Nut Consumption and Body Adiposity

Nut consumption may confer numerous health benefits, mainly because their antioxidant and anti-inflammatory properties are related to multiple potential mechanisms [6,14,17,18]. However, in the recent past, there was some apprehension that nuts could cause weight gain because of their high energetic value. Epidemiological evidence available in the literature are mainly related to cardiovascular disease, with a few studies which had outcomes of obesity and overweight. The Nurses’ Health Study (NHS) [3], which investigated 51,118 healthy, middle-aged women for 8 years, concluded that higher nut consumption was not associated with body weight gain. The results of the cited seminal cohort study suggested that adding nuts into diets does not lead to weight gain and may help to control weight. In accordance with the results of the NHS study, in a cross-sectional study with 803 adults, nut consumption was inversely associated with obesity and metabolic syndrome, regardless of demographic factors, lifestyle, and other dietary factors [5]. Recently, nut consumption was reported as a protective factor against metabolic syndrome, which is highly associated with obesity [4]. Besides indicating consumption higher than 5 servings per week of nuts as a protection factor, for each additional serving of walnuts consumed, the incidence of metabolic syndrome decreased by 3% (OR: 0.97; CI: 0.93–0.99), after adjusting for confounding factors [4]. Therefore, epidemiological studies demonstrate that nut consumption does not induce weight gain despite its high energy density, but protects against the development of several chronic diseases, including obesity.

This sequence of findings encouraged researchers to investigate the effects of nut consumption on body adiposity (reviewed in [6]). Table 1 summarizes these clinical findings. Randomized trials with positive effects on body mass were conducted in adults supplemented with 30 to 84 g of almond, macadamia, pistachio, or walnut, during 3 to 24 weeks. Regarding almonds, the effect on body weight loss was observed in 12 weeks with at least a daily consumption of 42 g [12]. The dose of walnut used was still lower than that of almonds (30 g) in the same period of intervention [7]. The study of Wien et al. [9] was carried out with the highest dose for relatively long period of intervention (84 g of almond, 24 weeks) and showed the best result on the body adiposity, compared to others presented in Table 1. In addition, the study conducted by Wu et al. [7] stands out because of its sample size, with 283 participants, and the study of Foster at al. [8] was carried out for 6 months, the longest time of intervention. Among the clinical trials reviewed in the literature, four are not described in Table 1 because the groups supplemented with nuts lost body weight, but with no differences compared to the control groups, although they showed an improvement in lipid profile and glucose metabolism [21,22,23,24]. The overall results of these trials indicate that nut consumption does not cause adverse effects on body weight or body composition, and even may aid the management of overweight and obesity.

Although the important details of the studies previously highlighted, trials using nuts as the only intervention strategy will be relevant to clarify the isolated effects of nut consumption on the management of overweight and obesity. In the trials reviewed, all participants received nutritional advice or physical activity intervention together with nut supplementation.

## 4. Nutritional Attributes and Bioactive Compounds of Nuts and Edible Seeds

Contents of nutrients and bioactive compounds of selected nuts and edible seeds are presented in Table 2, per serving of 1 ½ oz, or 42.5 g [25]. The selected nuts are the most commonly consumed worldwide and the most tested in clinical trials that studied the effect of nut consumption on body adiposity [6]. Table 2 also includes data for peanuts, the most usually consumed edible seed, and baru almond, an edible seed native to Brazil with great nutritional profile and high potential of health promotion [26].

Nuts and edible seeds have high energy density and high nutrient content with healthy profiles. Pecans showed the highest (around 300 kcal/serving) and baru almond the lowest (approximately 230 kcal/serving) energy concentration, compared to other selected nuts and edible seeds (Table 2). Lipid is the largest nutrient present in these foods, hence they are named oilseeds, but their total lipid contents and profiles differ considerably. Almond, cashew, pistachio, baru almond, and peanut featured the lowest amounts (18–19 g/serving), whereas Brazil nut, hazelnut, pecan, and walnut presented the highest lipid concentrations (26–28 g/serving). The main fatty acids of the almond, cashew, pecan, edible seed, and mostly hazelnut are monounsaturated fat acids (MUFA), and the Brazil nut, pistachio, and especially walnut contain mainly polyunsaturated fat acids (PUFA). The Brazil nut and cashew also have considerable content of saturated fatty acids (SFA). In addition, almond, hazelnut, and pecan showed the highest MUFA:SFA ratios (approximately 7:1–9:1). Monounsaturated fatty acids consumption is associated with lower risk for cardiometabolic disorders, such as dyslipidemia, obesity, and insulin resistance [27].

Besides lipids, oilseeds are source of good quality protein and dietary fiber. The protein content of nuts range from 3 to 8 g per serving, and those of the edible seeds are higher (13 g/serving) (Table 2). The essential amino acids profile of the oilseed proteins supplies the adult human requirement [15]. In vivo study showed that the Protein Digestibility-Corrected Amino Acid Score values for proteins from cashew nuts and peanuts were around 80%, and for baru almonds, 90% [28]. Additionally, the dietary fiber contents of the oilseeds are considerable, mainly of almond, hazelnut, pecan, pistachio, and baru almond (4–5 g/serving), and can contribute to increasing the fiber content of the diet. Therefore, these foods are an alternative to provide good-quality plant protein and lipid, and dietary fiber in healthy diets to reduce the risk of obesity and its comorbidities.

Concerning the lipophilic compounds of the oilseeds, tocopherols are found in high concentrations in almond, hazelnut, baru almond and peanut (Table 2), whose values are relevant when compared to the dietary reference intakes for vitamin E (α-tocopherol equivalents: 15 mg/day) [29]. In addition to its nutritional function, vitamin E features antioxidative, anti-inflammatory, and antiobesity properties [30]. Nuts and edible seeds are also sources of phytosterols, especially pistachio. Studies on food phytosterol composition are scarce in the literature, despite the importance of the topic. These compounds may inhibit the intestinal absorption of cholesterol and reduce the risk of hyperlipidemia [31,32], which is an important obesity comorbidity. Carotenoids, another lipophilic constituent of plant foods, are found in low amounts in nuts and edible seeds, except pistachio, which showed very high lutein content. Lutein is a bioactive compound with potent antioxidant activity, so it may help protect against chronic oxidative state present in obesity [33].

Phenolic compounds are the most abundant phytochemicals in the nuts and edible seeds. Pecan, pistachio, walnut, and baru almond present the highest values (around 300–700 mg/serving) among the selected nuts and edible seeds. Moreover, some oilseeds stand out for their high content of flavonoids and tannin (pecan), flavonoids (walnut), and tannins (baru almond). Other oilseeds are considerable sources of tannins, except Brazil nut and cashew (Table 2). Nevertheless, data about phenolic profiles of nuts and edible seeds are still limited and scarce. Flavonoids and tannins can attenuate the pro-oxidant and proinflammatory status and thus may decrease the risk of obesity and inflammatory diseases (reviewed in [18,34]).

## 5. Potential of Nut and Edible Seed Consumption on the Remodeling of Gut Microbiota

Diet is a key modulator of the gut microbiota, and there is a large body of evidence about the influence of different plant foods on the host microbiota composition. Dietary fiber (nondigestible polysaccharides) and polyphenols (polymerized compounds) are the main components of the plant foods responsible for their prebiotic properties [39].

Nuts and edible seeds are rich in complex polyphenols (mainly tannins) and dietary fiber (see Table 2), which have prebiotic effects in the host gut. The nondigestible polysaccharides (cellulose, hemicelluloses, pectin substances, etc.) are fermented by the intestinal bacteria to short-chain fatty acids (SCFA), especially butyrate [40,41]. Dietary polyphenols, in turn, are only partially absorbed in the small intestine during the digestion process. Complex polyphenols remain unabsorbed in the gut, and then they are bioactivated in the colon by the microbiota. The microbiota metabolites of these complex polyphenols are smaller molecules that are absorbed through the colon barrier [42]. Ellagitannins (hydrolysable tannins) and proanthocyanins (condensed tannins) are the main phenolic compounds of nuts and edible seeds. The active metabolites of nut polyphenols are mainly ellagic acid (and its metabolite urolithins) from ellagitannins [43], and valerolactones and phenolic acids from proanthocyanins [44]. These metabolites are found in the blood of the host and thus may have potential effects on human metabolism and health.

According to the results of in vitro and in vivo studies, overall prebiotic compounds of nuts may stimulate the growth of nonpathogenic gut bacterial species, and at the same time, inhibit the growth of pathogenic ones (see Table 3). In vitro studies showed the prebiotic effects of whole and defatted almonds [40], raw and roasted almonds [45], and fiber and extracts of chestnut [46]. In the study of Mandalari et al. [40], the potential on the positive remodeling of gut microbiota was confirmed by a higher prebiotic index of almonds than that of commercial prebiotic fructooligosaccharides. Prebiotic properties of raw and roasted almonds were confirmed in specific-pathogen-free rats [45]. In addition, 344 Fisher rats treated with walnuts (11% of the diet) for six weeks presented strong prebiotic effects by increasing different nonpathogenic bacterial species [47]. However, in a randomized, controlled, crossover trial, health adults treated with 1.5 and 3 servings (42.5 g and 85 g, respectively) per day of almonds for 18 days did not enhance their beneficial bacteria population [41]. Nevertheless, in the cited study, the treatment with pistachio increased the number of potentially beneficial butyrate-producing bacteria, but no effect was observed in *Lactobacillus* and *Bifidobacterium* species [41]. In another interventional study, the diet of healthy adult volunteers was supplemented with 56 g per day of roasted almonds or 10 g of almond skin for 42 days [48]. The prebiotic effects on *Lactobacillus* spp. and *Bifidobacterium* spp. observed in this study for almond and almond skin groups may be explained by the longer period of intervention compared to the Ukhanova et al. study [41]. In a recent randomized, controlled, crossover trial, Holscher et al. [49] showed that the processing of almonds affects the composition of the gastrointestinal microbiota, as the treatment for 21 days with 42 g per day of chopped almond or almond butter, but not with whole almond, enhanced the beneficial bacterial genera. Similarly, but over a longer period of supplementation (56 days), daily intake of walnuts (43 g) significantly affected the gut microbiome by enhancing probiotic and butyric acid-producing bacteria in healthy individuals [39].

In summary, the results of these studies suggest that the consumption of around 42.5 g for at least 3 weeks may modulate positively the host microbiota. However, more clinical trials over a longer time period with different servings are required to clarify the prebiotic effects of the consumption of nuts and edible seeds on intestinal bacteria.

To our knowledge, no clinical trial has been published about the effects of nuts on the remodeling of obese individuals’ microbiota. The profile of gut bacterial population seems to change between lean and obese individuals, although obesity is associated with different profiles of gut microbiota [50]. Therefore, human studies in this field will be relevant to understand the role of nut and edible seed consumption on body adiposity control, and the influence of positive remodeling of intestinal microbiota in this process.

## 6. Microbiota and Energy Metabolism: Dysbiosis and Intestinal Homeostasis

**Figure 1:**
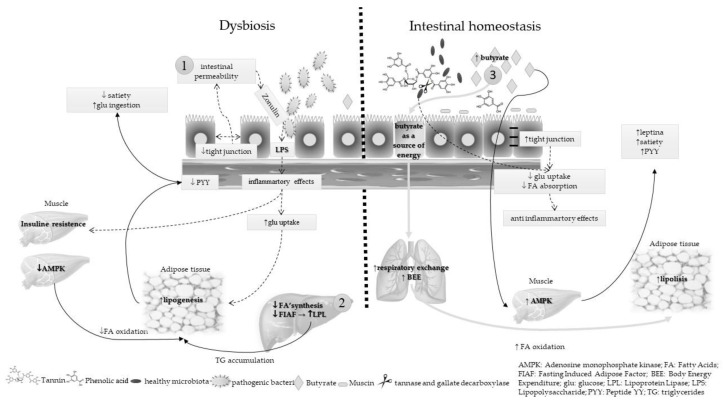
Energy metabolism modulation by microbiota: dysbiosis and intestinal homeostasis.

The microbiota plays an important role in energy metabolism, and thus may contribute to the incidence and prevalence of obesity through at least three pathways: increasing of intestinal permeability, enhancement of inflammatory status, and impairment of butyrate synthesis. Figure 1 summarizes these three plausible mechanisms and the contribution of nut and edible seed prebiotics to the intestinal homeostasis.

Intestinal permeability was evaluated by serum zonulin (one of the proteins responsible for the junction of enterocytes) concentration [51]. Previous studies demonstrated that zonulin is an important biomarker of chronic low-grade inflammation, which is directly associated with dysbiosis and intestinal permeability. The mechanism involved in this process is related to the metabolic endotoxemia caused by serum lipopolysaccharides (LPS). In addition to association with serum LPS and inflammatory markers, serum zonulin concentration was directly associated with markers of glucose and lipid metabolism, again indicating its role in increased intestinal permeability [51].

Low-grade inflammation in adipose tissue is an important component of the pathophysiology of insulin resistance in obesity. Microbial composition can affect the host metabolism, inducing systemic insulin resistance and modifying glucose homeostasis and the immune response. The modifications in the gut microbiota composition contribute to these metabolic changes, as demonstrated by the fact that some of the metabolic abnormalities associated with obesity can be reproduced in germ-free mice by colonization with gut microbiota from obese mice or obese humans [52]. In the same study, the treatment with prebiotics and probiotics reduced insulin resistance and inflammation in peripheral organs, such as liver, fat, and muscle, in mice models.

Butyrate, a short-chain fatty acid produced by microbiota from dietary fiber, can increase the adenosine monophosphate kinase (AMPK) activity in liver and muscle. AMPK is an enzyme that plays a role in cellular energy homeostasis and controls the activity of transcription factors involved in the cholesterol, lipid, and glucose metabolism. Butyrate activates AMPK by increasing the adenosine monophosphate/adenosine triphosphate (AMP/ATP) ratio. In vitro studies showed that butyrate increased the AMP/ATP ratio and AMPK activity in both muscle and liver cells in a leptin-dependent manner [53]. Leptin, an adipokine that regulates energy expenditure and food intake, stimulates fatty acid oxidation by increasing the AMP/ATP ratio and AMPK activity in liver and muscle. In addition, total body energy expenditure is increased with the increase of respiratory exchange ratio, indicating increased fatty acid oxidation. In low dietary fiber intake, the butyrate synthesis is impaired, which intensifies the obesity process and dysbiosis [53].

Nuts and edible seeds, which are rich in dietary fiber and polyphenols, mainly tannins (Table 2), contribute to the modulation of energy metabolism through the microbiota. Polyphenols can decrease the synthesis of fatty acids in the liver or delay their absorption in intestines. Polyphenols can also slow down digestion of carbohydrates through the inhibition of digestive enzymes or modulation of glucose uptake [54].

The hydrolysis of tannins to phenolic acids is catalyzed by the action of tannase and gallate decarboxylase. Some bacterial species, including *Lactobacillus plantarum*, *L. paraplantarum*, and *L. pentosus*, express these enzymes and promote the intestinal bioactivation of polyphenols [55]. Finally, the gut microbiota plays an essential role in metabolism of glucose, and thus can impact the development and treatment of obesity.

Three mechanisms are proposed to explain the energy metabolism modulation by microbiota during dysbiosis and the role of nut and edible seed prebiotics in maintaining the intestinal balance:

Arrow 1—in dysbiosis, there is a loss of tight junctions with consequent withdrawal of enterocytes and increased intestinal permeability. In this way, membrane lipopolysaccharides (LPS) of gram-negative bacteria penetrate the intestinal barrier and reach the blood circulation. The presence of LPS in the bloodstream is a proinflammatory factor, which induces insulin resistance in muscle and increases adipogenesis. On the other hand, in intestinal homeostasis, the dietary fiber and polyphenol consumption stimulate the proliferation of beneficial bacteria in the intestine, as well as the butyrate and mucin production, which maintains the integrity of the enteric barrier. Thus, the microbiota exerts an anti-inflammatory effect and the glucose and fatty acids uptakes are decreased.

Arrow 2—in dysbiosis, adenosine monophosphate kinase (AMPK), an enzyme expressed mainly in the muscle, is inhibited by gut microbiota. It negatively influences fatty acid oxidation, promotes the synthesis of cholesterol and triglycerides (TG), and favors lipogenesis. Furthermore, fasting-induced adipose factor (FIAF) is involved in energy metabolism as an adiposity regulator by the inhibition of lipoprotein lipase (LPL), which in turn enhances TG accumulation in adipocytes. The transferred gut microbiota suppresses the FIAF expression in intestinal epithelium that, in turn, causes enhanced fatty acid uptake by adipocytes via increased LPL activity. In intestinal homeostasis, butyrate increases the AMPK activity. It is important in regulation of cholesterol, lipid, and glucose metabolisms, because the fatty acid oxidation is enhanced in muscle and adipose tissue. Butyrate also regulates food intake and satiety via modulation of intestinal enteroendocrine L cells derived from peptides, mainly GLP1 and peptide YY (PYY). The function of PYY is to reduce appetite by acting upon neuropeptide Y (NPY), thereby inhibiting gastric motility and reducing food intake.

Arrow 3—in intestinal equilibrium, the production of butyrate is high. This short-chain fatty acid is a source of energy for the enterocytes, which increases the total body energy expenditure (BEE) along with a decreased respiratory exchange ratio, indicating increased fatty acid oxidation. In addition, the plasma leptin level is increased, which also helps to increase the satiety and decrease the glucose uptake.

## 7. Conclusions and Future Perspectives

Nut and edible seed consumption have numerous benefits for human health, particularly related to prebiotic effects on gut microbiota derivate from their high contents of dietary fiber and polyphenols. Therefore, the potential effect of nuts and edible seeds on the prevention and management of obesity seems to be associated with their prebiotic properties. Three plausible mechanisms are proposed in this review to explain the potential benefits of nut and edible seed consumption on gut microbiota and their relationship to the body adiposity control. Human trials on the prebiotic properties of nuts and edible seeds are still limited, especially with overweight and obese participants, and the following mechanisms proposed should be investigated in this context: 

maintaining of the enteric barrier integrity, improvement of inflammation status and enhancement of butyrate synthesis.

Moreover, few species of intestinal bacteria responsible for polyphenols activation have been identified, and there is little knowledge about the mechanisms involved. Further investigations should explore the interaction between dietary fiber and polyphenols metabolites from nuts and their relation to specific gut bacteria, as well as the related mechanisms that contribute to the host health, especially in overweight and obese individuals supplemented with different types of oilseeds.

## Figures and Tables

**Table 1:** Summary of clinical trials about the effects of nut consumption on body adiposity.

Reference	Sample	Study Design	Study Findings	
		Nut/Edible Seed (Dose Daily, Time of Intervention)	Significant Result	*p* Value
Wu et al. 2010 [7]	283 adults with metabolic syndrome	Walnut (30 g, 12 weeks)	⇓ waist circumference	<0.05
Foster et al. 2012 [8]	123 overweight and obese adults	Almond (56 g, 6 months)	⇓ body weight	0.04
Wien et al. 2013 [9]	65 overweight and obese adults	Almond (84 g, 24 weeks)	⇓ body weight	<0.05
			⇓ waist circumference	
			⇓ body fat	
Somerset et al. 2013 [10]	64 overweight and obese adults	Macadamia (50% E from fat as MUFA,~50 g, 10 weeks)	⇓ waist circumference	<0.05
Gulati et al. 2014 [11]	60 adults with metabolic syndrome	Pistachio (20% E, ~50 g, 3 weeks)	⇓ waist circumference	0.02
Dhillon et al. 2016 [12]	86 overweight and obese adults	Almond (42 g, 12 weeks)	⇓ body fat	0.04

50% E, percentage of the diet energy from fat; ⇓, decreased; MUFA, monounsaturated fatty acids.

**Table 2:** Nutrients and bioactive compounds of selected nuts and edible seeds per serving of 42.5 g.

Nutrients and Bioactive Compounds		Nuts							Edible Seeds		References
		Almond	Brazil Nut	Cashew	Hazelnut	Pecan	Pistachio	Walnut	Baru Almond	Peanut	
Energy (kcal)		246	284	244	275	302	242	278	232	250	[28,35]
Total protein (g)		8.28	5.92	7.99	5.98	3.19	8.42	5.72	12.72	12.58	[15,28]
Lipid (g)	Total	18.43	28.35	18.58	26.12	28.13	19.16	27.41	18.14	18.72	[15,36]
Fat acids (g/100 g oil)	SFA	9.09	25.35	21.12	9.11	8.35	14.24	11.76	15.47	19.37	[15,36]
	PUFA	29.31	45.61	17.19	7.79	24.92	51.47	72.96	31.71	37.76	[15,36]
	MUFA	61.60	29.04	61.18	83.10	66.73	34.29	15.28	51.57	42.72	[15,36]
	MUFA/SFA	6.7	1.1	2.9	9.1	8.0	2.4	1.3	3.3	2.2	[15,36]
Dietary fiber (g)	Total	5.31	2.85	1.28	4.00	4.00	4.38	3.02	3.91	2.21	[28,35]
	Soluble	-	-	-	-	-	-	-	0.86	0.58	[28]
	Insoluble	-	-	-	-	-	-	-	3.05	1.63	[28]
α-TE (mg/100 g oil)		25.0	4.3	1.3	33.1	3.7	7.3	5.5	11.6	11.6	[16,37]
Phytosterols (mg/100 g oil)		218	193	199	110	196	559	124	-	173	[17]
Carotenoids (µg)	Total	ND	ND	ND	ND	ND	2040	ND	5	-	[16,37]
	Lutein	ND	ND	ND	ND	ND	1870	ND	-	ND	[16]
	β-carotene	ND	ND	ND	ND	ND	170	ND	-	ND	[16]
Phenolics (mg)	Total (range)	102 (55–194)	48 (43–57)	58 (56–60)	124 (43–184)	546 (434–614)	369 (209–613)	691 (444–872)	309	179 (139–235)	[16,38]
	Flavonoids	40	46	27	49	300	61	317	-	62	[17]
	Tannin	123	4	17	98	374	94	145	239	-	[15,37]

SFA, Saturated Fatty Acids; PUFA, Polyunsaturated Fatty Acids; MUFA, Monounsaturated Fatty Acids; MUFA/SFA, MUFA/SFA ratio; α-TE, α-tocopherol equivalents; ND, Not detected.

**Table 3:** Effect of nuts on the remodeling of gut microbiota in vitro, in animal models, and in human studies.

Reference	Aim	Intervention	Study Type	Study Design	Study Findings
Mandalari et al. 2008 [40]	To investigate the potential prebiotic effect of almonds in vitro by using mixed fecal bacterial cultures	Finely ground whole (FG) and defatted (DG) almonds	In vitro	In vitro gastric and duodenal digestion of the almond samples and fractions were subsequently used as substrates for the colonic model in which the composition and metabolic activity of gut bacteria populations were evaluated	Whole almond: *Bifidobacteria* ⇑ *Eubacterium rectale* and butyrate production ⇑
Blaiotta et al. 2013 [46]	To evaluate chestnut components as probiotic carriers by examining the effect on the viability of selected lactic acid bacteria (LAB)	Chestnut extracts and chestnut fiber (LAB viability during 180 min)	In vitro	Simulated gastric (with pepsin) and bile (with pancreatin) juices were prepared and added to cultured LAB cells (12 strains) with chestnut fiber or chestnut extracts	*Lactobacillus paracasei* GG ⇑ *Lactobacillus rhamnosus* ⇑ *Lactobacillus casei* ⇑ *Streptococcus macedonicus* ⇓ *Streptococcus thermophilus* ⇓
Liu et al. 2016 [45]	To compare the fermentation properties of raw and roasted almonds	Predigested raw and roasted almonds (0, 2.5, 5, 10, 15%)	In vitro	Hydrolyzed raw and roasted almonds under simulated gastric and duodenal digestion were added to cultured *Lactobacillus acidophilus, Bifidobacterium breve*, and *Escherichia coli* and incubated anaerobically at 37° for 48 h	*Lactobacillus acidophilus* ⇑ *Bifidobacterium breve* ⇑ *Escherichia coli* ⇓
Liu et al. 2016 [45]	To test the prebiotic effect of raw and roasted almonds on faecal and caecal bacteria	Raw and roasted almonds (5 g/kg BW - 1 g/day for 4 weeks via intragastric	Animal model	Male specific-pathogen-free (SPF) Wistar rats (30) with 10-week-old. They were randomly divided into three groups (10 rats per group) according to feeding regime: control, raw almonds, and roasted almonds	*Lactobacillus* ssp. ⇑ *Bifidobacterium* ssp. ⇑ *Enteroccocus* ssp. ⇓ *Escherichia coli* ⇓
Byerley et al. 2017 [47]	To investigate if walnuts modulate the gut microbiome and promote their health benefit	Walnuts (approximately 1.7 g/day for 6 or 10 weeks)	Animal model	Male Fischer 344 rats (20) in two groups: 1) control diet, 2) walnut diet, with 11% walnuts replacing protein (casein), fat (oil), and fiber (cellulose) of the control diet. Fecal samples were collected from descending colon at the sacrifice	*Lactobacillus* ⇑ *Ruminococcaceae* ⇑ *Roseburia* ⇑ *Bacteroides* ⇓ *Anaerotruncus* ⇓ *Alphaproteobacteria* ⇓
Ukhanova et al. 2014 [41]	To evaluate if intake of nuts affects bacterial or fungal microbiota composition	Almond (0, 1.5 and 3 servings/day: 0, 42.5 or 85 g for 18 days each treatment)	Randomized, controlled, crossover trial	Healthy adults (*n* = 18) with three 18 d feeding periods separated by a washout period of 2 weeks. During the 3 treatment periods, the same-base typical low-fiber American diet was provided	*Lactobacillus* ⇒ *Bifidobacteria* ⇒
		Pistachio (0, 42.5 or 85g/day for 18 days each treatment)		Healthy adults (*n* = 16) with three 18 d feeding periods separated by a washout period of 2 weeks. The provided diet was the same for almond treatment	Butyrate-producing bacteria ⇑ *Bifidobacteria* ⇒ *Lactobacillus* ⇒ lactic acid bacteria ⇓
Liu et al. 2014 [48]	To investigate the prebiotic effects of almond and almond skin intake in healthy humans	Roasted almonds (56 g/day) and almond skin (10 g/day) for 6 weeks	Randomized, controlled trial	Healthy adult volunteers (*n* = 48, 16 for each treatment) consumed almonds (56 g), almond skin (10 g), or commercial fructooligosaccharides (8 g) (as positive control) daily for 6 weeks. Diet was provided by the school canteen, which excluded peanuts or other nuts	*Lactobacillus* ssp. ⇑ *Bifidobacterium* ssp. ⇑ *Escherichia coli* ⇒ *Clostridum perfringens* ⇓
Holscher et al. 2018 [49]	To assess the interrelationship of almond consumption and processing on the gastrointestinal microbiota(bacterial genera)	Whole almonds (WA); whole, roasted almonds; roasted, chopped almonds (CA), and almond butter (42 g)/day for 21 days each treatment)	Randomized, controlled, crossover trial	Healthy adults (*n* = 18), controlled-feeding, five periods of 3 weeks, crossover study with washouts between diet periods was conducted. Treatments included: (1) zero servings/day of almonds (control); (2) 1.5 servings (42 g)/day of whole almonds; (3) 1.5 servings/day of whole, roasted almonds; (4) 1.5 servings/day of roasted, chopped almonds; and (5) 1.5 servings/day of almond butter	CA: *Lachnospira* ⇑ *Roseburia* ⇑ *Oscillospira* ⇑ WA: *Dialister* ⇑
Bamberger et al. 2018 [39]	To investigate the effect of walnut intake on the gut microbiome composition and microbial diversity	Walnut (43 g/day) for 8 weeks	Randomized, controlled, crossover trial	Healthy nonsmoking subjects (*n* = 135) older than 50 years (men and postmenopausal women) subjects were randomized to 2 different diet phases, walnut-enriched diet (43 g/day) or nut-free control diet, for 8 weeks (separated by a 4-week washout)	Butyrate-producing bacteria ⇑ *Ruminococcaceae* ⇑ *Bifidobacteria* ⇑ *Clostridium* sp. ⇓

⇑, increased; ⇒, maintained; ⇓, decreased; BW, body weight.
